# Broadband High-Efficiency Grating Couplers for Perfectly Vertical Fiber-to-Chip Coupling Enhanced by Fabry-Perot-like Cavity

**DOI:** 10.3390/mi11090859

**Published:** 2020-09-17

**Authors:** Zan Zhang, Beiju Huang, Zanyun Zhang, Chuantong Cheng, Bing Bai, Tianxi Gao, Xiaobo Xu, Wenping Gu, Lin Zhang, Hongda Chen

**Affiliations:** 1 School of Electronics and Control Engineering, Chang’an University, Xi’an 710064, China; baibing@chd.edu.cn (B.B.); tianxigao@chd.edu.cn (T.G.); xuxiaobo@chd.edu.cn (X.X.); wpgu@chd.edu.cn (W.G.); zhanglin_dk@chd.edu.cn (L.Z.); 2 State Key Laboratory on Integrated Optoelectronics, Institute of Semiconductors, Chinese Academy of Sciences, Beijing 100083, China; bjhuang@semi.ac.cn (B.H.); chengchuantong@semi.ac.cn (C.C.); hdchen@semi.ac.cn (H.C.); 3 School of Electronics and Information Engineering, Tianjin Polytechnic University, Tianjin 300387, China; zhangzanyun@tjpu.edu.cn

**Keywords:** silicon photonics, grating coupler, Fabry-Perot cavity, photonic integrated circuit

## Abstract

We propose a broadband high-efficiency grating coupler for perfectly vertical fiber-to-chip coupling. The up-reflection is reduced, hence enhanced coupling efficiency is achieved with the help of a Fabry-Perot-like cavity composed of a silicon nitride reflector and the grating itself. With the theory of the Fabry-Perot cavity, the dimensional parameters of the coupler are investigated. With the optimized parameters, up-reflection in the C-band is reduced from 10.6% to 5%, resulting in an enhanced coupling efficiency of 80.3%, with a 1-dB bandwidth of 58 nm, which covers the entire C-band. The minimum feature size of the proposed structure is over 219 nm, which makes our design easy to fabricate through 248 nm deep-UV lithography, and lowers the fabrication cost. The proposed design has potential in efficient and fabrication-tolerant interfacing applications, between off-chip light sources and integrated chips that can be mass-produced.

## 1. Introduction

Benefitting from mature complementary metal-oxide-semiconductor (CMOS) technology, various photonic devices have been demonstrated based on low loss silicon-on-insulator (SOI) waveguides, showing great promise for electronic-photonic integrated circuits, high-density photonic integrated circuits (PICs), and three-dimensional (3D) photonic integration [1,2,3,4,5]. Grating couplers (GCs) enable optical coupling between standard single-mode optical fiber and high index contrast SOI waveguides, and have gained widespread usage as fiber-to-chip couplers for SOI PICs [6,7,8]. Compared to edge couplers, GCs enable position-friendly interfacing of silicon chips by optical fibers so that wafer-scale optical measurements can be performed without dicing [8,9].

In the past, much effort has been devoted to the design and optimization of GCs [10,11,12,13] for interfacing between single mode fiber and silicon photonic chips. However, off-normal GCs couple light from tilted fiber (usually ~10°), to avoid second-order reflection [10], which is disadvantageous for rapid wafer-scale tests and low-cost photonic packaging [8]. Therefore, perfectly vertical GCs are appealing, as they facilitate easier alignment and packaging processes.

Perfectly vertical GCs, with increased coupling efficiency, (CE) have been widely explored over the past decade. Various approaches have been proposed, including employing slanted grating [14], chirping the GC [15,16,17], relying on a tilted silicon membrane structure [18,19], adding an anti-back reflection structure [20,21,22], and employing a dual-layer grating structure [23,24,25]. However, the above reported high-efficiency approaches require either extra fabrication processes or sophisticated device structure, with a minimum feature size below 200 nm, which increases the fabrication cost drastically, and affects the fabrication error tolerance.

In this work, by taking advantage of silicon nitride (Si_3_N_4_) material and the idea of Si-overlay for a GC, we present a broadband high-efficiency bidirectional perfectly vertical GC, with numerical demonstration. The bidirectional GC functions as both a fiber coupler and a 3-dB optical power splitter, so that it can, not only act as a highly efficient fiber-to-chip coupler, but also work in Mach–Zehnder interferometer based optical components [26,27]. Si-overlay on GC is utilized to increase directionality [7]. A Fabry-Perot-like cavity, composed of the Si_3_N_4_ layer and the grating itself, is introduced into the coupler to suppress the light back-reflected into the launching fiber, thus enhancing the coupling into the on-chip waveguides. Numerical calculations based on the finite-difference time-domain (FDTD) were implemented to determine the dimensional parameters. With the help of the Fabry-Perot-like cavity, reflection back towards the fiber is decreased to 5%, and an enhanced CE of 80.3% is achieved. In addition, since there are fewer grating periods than most other designs, the proposed GC shows a 58 nm-wide 1-dB bandwidth that covers the whole C-band, which is favorable for wide band operation. The minimum line width of the proposed GC is larger than 219 nm, which will lower the fabrication cost and improve the fabrication tolerance.

**Figure 1:**
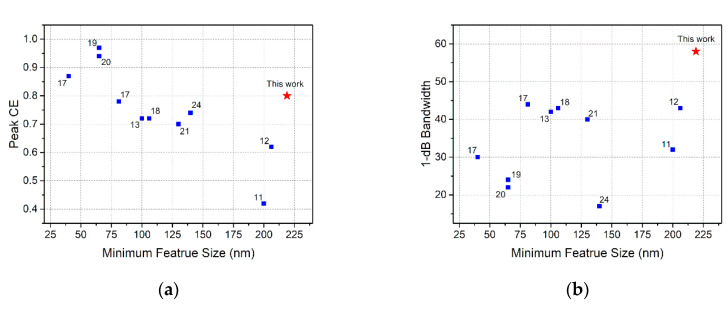
Comparison of our perfectly vertical grating coupler (GC) simulation result, with previously reported works with different minimum feature size: (**a**) coupling efficiency; (**b**) 1-dB bandwidth. The numbers next to the markers are the references.

In Figure 1, our simulated result is plotted next to a summary of perfectly vertical GC demonstrations; the numbers next to the markers indicate the references. As shown in Figure 1a, we have achieved competitive high CE with the largest feature size. The 1-dB bandwidth demonstrated here is a record among high-efficiency GCs, shown in Figure 1b. Such a design can provide efficient, robust, and cost-effective coupling interfaces for sub-micrometric SOI waveguides, as desired for silicon PIC packaging, with fiber or integrating with VCSLE.

## 2. Device Structure and Principle

**Figure 2:**
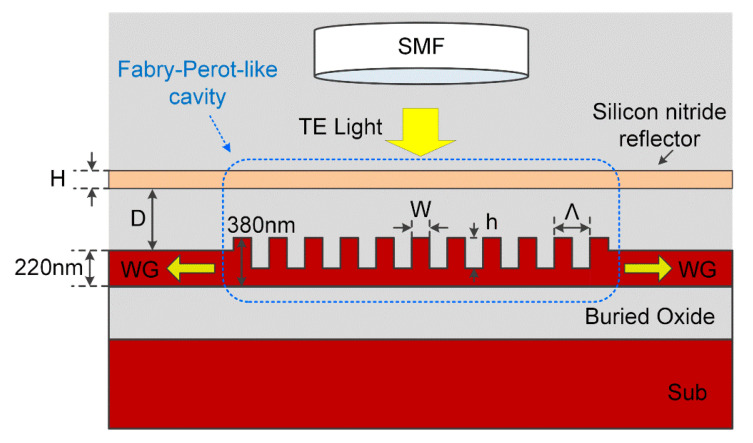
Schematic diagram of the device configuration. The Si_3_N_4_ layer and the grating, together form a Fabry-Perot-like cavity to reduce the up-reflection, thus enhancing the coupling efficiency (CE).

Figure 2 shows our proposed perfectly vertical silicon fiber-to-chip GC. The proposed structure is a so-called bi-directional GC, which is based on a uniform Si-overlay grating, with two opposite in-plane transmission ports. Si-overlay is employed here to improve the vertical asymmetry of the grating, in order to achieve high directionality (defined as the ratio of power diffracted upward to the total diffracted optical power), and thus coupling efficiency. Differently from off-normal GCs, the period Λ of the proposed grating equals the wavelength divided by the effective refractive index *n*_eff_, so that the first-order diffraction couples light out of the waveguide, producing a surface-normal propagating field, to achieve perfectly vertical coupling [28]. One may argue that, with this typical Λ described above, the uniform grating is under resonance statues, which is commonly unwelcome in most coupler applications. Considering chip-to-fiber coupling, the second-order diffracted mode will cause a strong reflection back into the waveguide. However, thanks to the symmetry of the bi-directional GC, with two in-plane ports, the reflected wave in one in-plane port will be diminished by the destructive interference, with the wave transmitted from the other in-plane port, as discussed in our previous work [29].

For coupling from fiber to chip, a light wave from a perfectly vertical single mode fiber is launched to the center of the uniform grating and coupled into two SOI waveguides, on both sides of the grating. The total CE for perfectly vertical in-plane coupling is mainly affected by up-reflection and substrate leakage. Up-reflected light, towards the launching fiber, attracts more concern in perfectly vertical coupling schemes, as it can significantly deteriorate the operation of the out-of-plane active launching element.

Most of the reported structures so far have studied the back-reflection to the waveguide, when the GC is used as the chip-to-fiber coupler. When the GC is used as the incoming coupler to the chip, as will be the case for most VCSEL to SOI assembly, there is little discussion on the up-reflection to the fiber. To reduce the up-reflection, and thus enhancing the whole CE, we introduced a Fabry-Perot-like cavity by adding a Si_3_N_4_ layer over the grating. Si_3_N_4_ is a dielectric commonly used as the passivation layer in back-end-of-line in the CMOS process, which ensures our design is compatible with CMOS process. The Si_3_N_4_ layer, and the grating itself, both work as reflective surfaces of the cavity. When the cavity is under resonance, the reflection of the whole cavity will reach the minimum value. The reflection of an asymmetric Fabry-Perot cavity can be obtained by [30]:

(1) $$R_{\mathrm{cavity}}=\frac{{\left(\sqrt{R_{1}}-\sqrt{R_{2}}\right)}^{2}+4\sqrt{R_{1}}\sqrt{R_{2}}\sin^{2}\left(\varphi \right)}{{\left(1-\sqrt{R_{1}}\sqrt{R_{2}}\right)}^{2}+4\sqrt{R_{1}}\sqrt{R_{2}}\sin^{2}\left(\varphi \right)}$$

where *R*_1_ and *R*_2_ are the reflectivity of the two reflective surfaces, and the phase *φ* is given by:

(2) $$2\varphi =\frac{4\pi nD}{\lambda }+\rho_{1}+\rho_{2}$$

where *n* is the index of refraction of the cavity medium, *D* is the cavity spacing, and *ρ*_1_ and *ρ*_2_ are the phase shifts of the two reflecting surface, respectively. Apparently, *R*_cavity_ is a periodic function of the cavity spacing *D*. Note that, zero reflection is possible, only when *R*_1_ = *R*_2_ and *φ* = *m*π (*m* is an integer), according to Equations (1) and (2). In other words, the closer *R*_1_ is to *R*_2_, the less reflection there will be for the Fabry-Perot cavity around the resonance wavelength.

In order to obtain a high-efficiency vertical GC, with zero up-reflection at the target wavelength, one should introduce a reflective surface with the same reflection characteristic of the grating, to form a symmetric Fabry-Perot cavity, and carefully design the distance between the reflector and the grating, to ensure the cavity is resonant at the desired wavelength. However, the reflection coefficient of the grating varies with the wavelength, moreover, the up-reflected wave of the grating cannot be seen as a plane wave, since the length of the grating is comparable to the mode size of the incident wave. Hence, if one want to achieve exactly the same reflection characteristics of the grating, a delicate design of reflector, with a complicated structure, might be needed, which will lead to extra fabrication processes and higher cost.

Therefore, considering the balance between the fabrication cost and the device performance, we employed a simple layer of Si_3_N_4_, a commonly used dielectric in CMOS technology, to act as a reflector, with a similar reflection coefficient to the grating at the desired wavelength, as shown in Figure 2. The Si_3_N_4_ layer and the grating together form a Fabry-Perot-like cavity, and obtain a reduced reflection around the resonant wavelength.

For the Si_3_N_4_ reflector, the reflection characteristic can be modeled as a symmetric Fabry-Perot cavity, so that the reflection coefficient and the phase shift of the reflector are written as:

(3) $$R_{1}=\frac{4R_{SiN}\sin^{2}\left(\varphi_{SiN}\right)}{{\left(1-R_{SiN}\right)}^{2}+4R_{SiN}\sin^{2}\left(\varphi_{SiN}\right)}$$

(4) $$\rho_{1}=\arctan\frac{\left(1-R_{SiN}\right)\sin2\varphi_{SiN}}{\left(1+R_{SiN}\right)\left(1-\cos2\varphi_{SiN}\right)}$$

where *R_SiN_* is the reflection coefficient of the Si_3_N_4_/OX interface which is 0.02541, and *φ_SiN_* is the one-pass phase shift in the Si_3_N_4_ layer, which is given by $\varphi_{SiN}=\frac{2\pi nH}{\lambda }$, where *H* is the thickness of the Si_3_N_4_ layer. Obviously, *R*_1_ and *ρ*_1_ are both functions of the thickness of the Si_3_N_4_ layer. Combined with the Equations (1)–(4), as long as *D* and *H* are carefully designed to make sure that *R*_1_ = *R*_2_ and *φ* = *m*π for the desired wavelength, the Fabry-Perot-like cavity will be under resonance, hence reduced reflection and enhanced CE will be achieved.

## 3. Design and Optimization

Our proposed coupling structure shown in Figure 2 is investigated in a commercial Finite-difference Time-domain (FDTD) solver (FDTD Solutions, Lumerical Inc., Vancouver, BC, Canada). The goal of the simulation is to obtain the highest CE for TE polarized light near 1550 nm. The width of the SOI waveguide is much larger than the height, so all of the simulations are two-dimensional. Table 1 shows the main parameters used in simulations. A TE polarized Gaussian beam with 1/e full width of 10.4 µm was launched onto the corrugated surface, and the coupling efficiency to the fundamental TE mode was examined. The proposed GC structures are based on an SOI substrate (shown in Figure 2), consisting of a standard 2 μm thick buried oxide (BOX) and a 220 nm thick top silicon layer, with a 160 nm thick Si-overlay; while the grating groove depth *h* is 230 nm, according to the *IMEC* MPW [31]. The values of the grating period, Λ, and filling-factor (FF = W/Λ, where W is the grating teeth width) are varied to optimize the fiber-to-chip CE. Notice that, due to the reciprocity of this coupling structure, the same CE is expected for the chip-to-fiber coupling.

**Figure 3:**
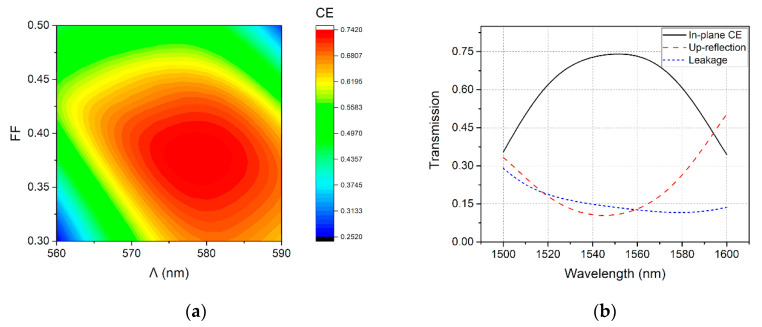
(**a**) The CE at 1550 nm with different Λ and filling factor (FF). The grating coupler with Λ = 578 nm and FF = 0.38 achieves CE of 74%. (**b**) Calculated transmission spectra of the Si-overlay grating coupler with Λ = 578 nm and FF = 0.38.

Figure 3a shows the obtained contour for the CE at 1550 nm, when Λ is varied from 560 nm to 590 nm and FF is varied from 30% to 50%. When the grating structure has a Λ ~578 nm and a FF ~38%, the CE can reach a maximum value of nearly 74%. Figure 3b shows the wavelength-dependent CE for in-plane coupling, up-reflection, and substrate leakage, for an optimized grating structure, with a Λ of 578 nm and an FF of 38%, determined from Figure 3a. Clearly, although the peak CE is as high as 74% at 1551 nm, there is about a quarter of the incident optical power, either coupled to the substrate, or reflected back to fiber. The up-reflection power is so high that the return loss reaches −9.7 dB. High return loss will induce damage to the off-chip light source, which is unacceptable in a practical system.

**Figure 4:**
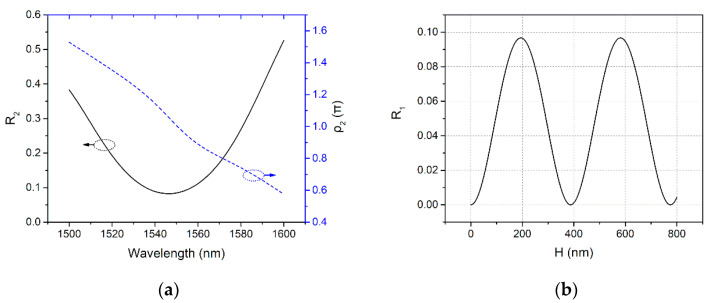
(**a**) Calculated *R*_2_ and *ρ*_2_ as a function of wavelength; (**b**) Calculated *R*_1_ with various *H*.

With the optimized grating period and filling-factor, we did a series of simulations to investigate the reflection characteristic of the GC. A field power monitor was positioned just above the grating and the light source to calculate the reflection coefficient and phase shift of the grating, *R*_2_ and *ρ*_2_, respectively. The length of the monitor was the same as the length of the grating to examine the up-reflected wave that resonated in the cavity. The optical path length accumulated from the light source to the field power monitor was taken into account for the calculation of *ρ*_2_. Figure 4a shows the calculated *R*_2_ and *ρ*_2_ as a function of wavelength. At 1550 nm, we obtained that *R*_2_ = 0.0847 and *ρ*_2_ = 0.975π. According to Equation (3), the corresponding thickness for the Si_3_N_4_ reflector could be obtained. It is worth noting that *R*_1_ is a periodic function of *H* as shown in Figure 4b. Here we chose the smallest value for *H,* which is 148 nm, since the longer the cavity length is, the higher the quality factor of a Fabry-Perot cavity will be [30], which is unwelcome in a wide-bandwidth application, such as optical coupling. With *H* = 148 nm, phase shift of the reflector *ρ*_1_ is obtained as 0.115π according to Equation (4). Combined with Equation (2) while *m* = 1 is assumed, *D* is obtained as 243 nm. Here we set *m* = 1, to get the smallest value of *D*, because a lower Q is favorable, as in the case of the value of *H*.

**Figure 5:**
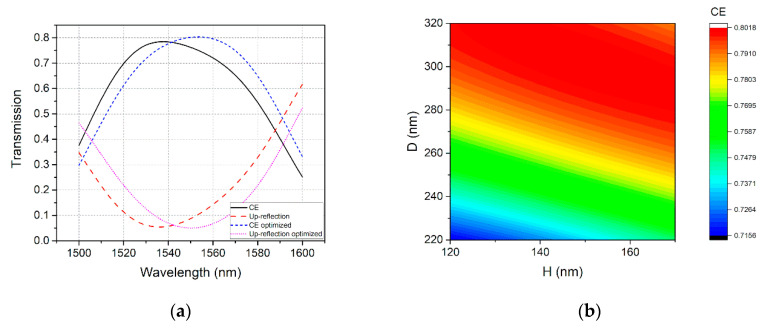
(**a**) Calculated transmission spectra of the cavity enhanced GC. (**b**) Calculated CE with *D* varying from 220 nm to 320 nm and *H* varying from 120 nm to 170 nm.

2D FDTD simulations were carried out to verify the performance of the GC, enhanced by the Fabry-Perot-like cavity. As depicted in Figure 5a, the peak CE was enhance to 78.5% at 1537 nm and the up-reflection was reduced to 5.4%, on account of the cavity. The values for *D* and *H* were swept in the simulations to verify if the optimal parameters were obtained. Figure 5b shows the contour for the CE at 1550 nm, when *D* is varied from 220 nm to 320 nm and *H* is varied from 120 nm to 170 nm. As can been seen, the optimal values for *D* and *H* are 302 nm and 149 nm respectively, which is a little different from the result calculated from the model discussed above. The reason for this difference may be that the profiles of the reflected fields of the grating and the Si_3_N_4_ reflector are not identical. Therefore, although the phase condition is met, completely destructive interference will not occur. Moreover, the reflection from the silicon substrate may affect the performance of the cavity as well. With the optimized *D* = 302 nm and *H* = 149 nm, the CE and up-reflection were calculated, as shown in short-dash curve and dot curve in Figure 5a, respectively. Thanks to the cavity, the peak CE reached 80.3% at 1551 nm and up-reflection was reduced to 5%.

**Figure 6:**
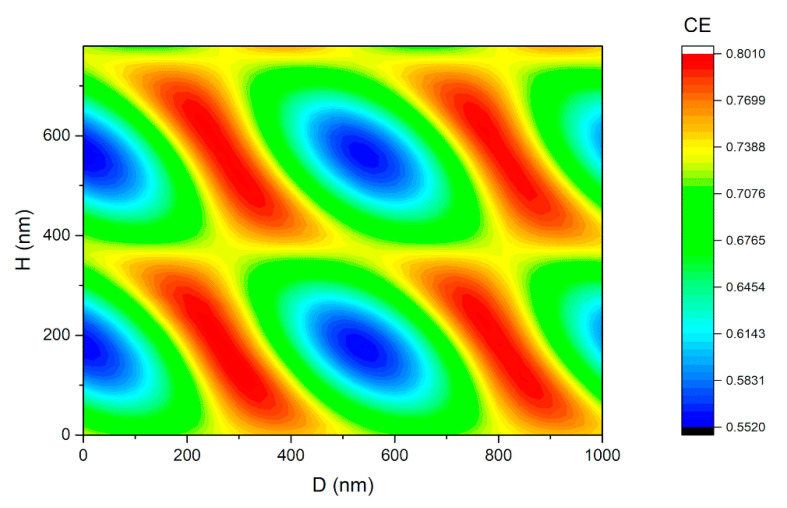
The CE at 1550 nm with *H* varying from 20 nm to 800 nm and *D* varying from 0 nm to 1000 nm. The CE changes periodically with the value of *H* and *D*.

**Figure 7:**
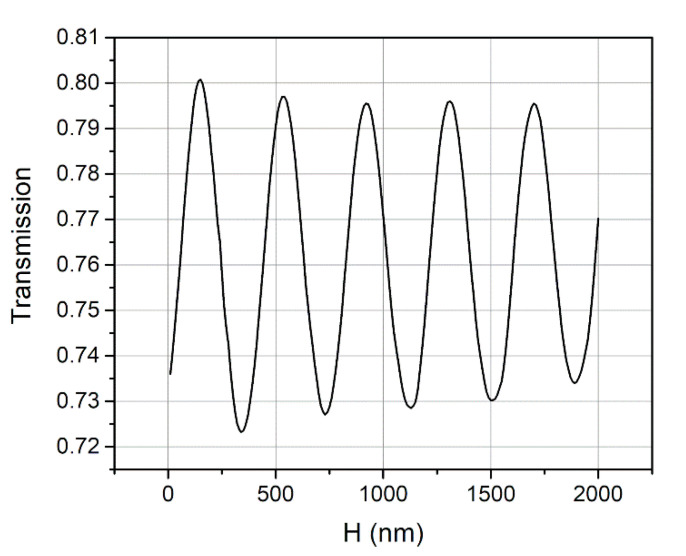
Calculated CE at 1550 nm with *D* = 302 nm and varying *H*. The peak value of the CE is slowly decreasing while *H* is increasing.

As we have discussed above, *R*_cavity_ varies periodically with *H* and *D*, leading to periodic relationships between enhanced CE and these two parameters. In order to further confirm whether *H* = 149 nm and *D* = 302 nm are optimal, a wide range parameter sweep was carried out through a series of simulations. Figure 6 shows the contour for the CE at 1550 nm obtained from the simulations, with *H* varying from 20 nm to 800 nm and *D* varying from 0 nm to 1000 nm. As clearly depicted, the CE changes periodically, with the value of *H* and *D*, and peak CE occurs with *H* = 149 nm and *D* = 302 nm. It is worth noting that, the peak value of the CE in every period is slowly decreasing while *H* and *D* are increasing. In order to show the decrease more clearly, the calculated CE, with *D* = 302 nm and varying *H,* is plotted in Figure 7. This decrease of CE was mainly because a small amount of the light power leaked from both sides of the Fabri-Perot-like cavity, along the horizontal direction. With larger values of *H* and *D*, more light leaks from the cavity, resulting in the decrease of the whole CE.

## 4. Discussion

### 4.1. Fabrication Process and Tolerance

**Figure 8:**
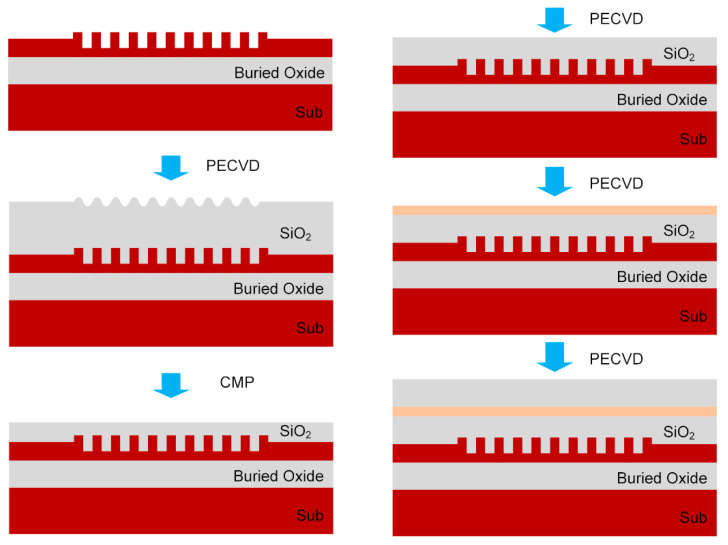
Process flow of the Si_3_N_4_ reflector over the grating for the Fabry-Perot-like cavity.

The grating structure with silicon overlay can be fabricated through polysilicon deposition, lithography, and etching based on SOI wafers with 220 nm thick silicon, as described in ref [7]. After the grating structure was accomplished, SiO_2_ was deposited by Plasma Enhanced Chemical Vapor Deposition (PECVD) to cover the grating, following chemical-mechanical polishing (CMP) to get a planarized surface. Then additional deposition of SiO_2_ was needed to achieve the certain thickness of SiO_2_ we desired. After that, Si_3_N_4_ layer was deposited through PECVD to form the upper reflector of the Fabry-Perot-like cavity. Finally, SiO_2_ was deposited, acting as a passivation layer. The fabrication process flow of the Si_3_N_4_ reflector over the grating is illustrated in Figure 8. As depicted in [33,34,35], 3D vertical integration of photonics devices is achievable through CMP and PECVD processes, with root-mean-square roughness of the SiO_2_ surface below 5 nm. Moreover, the thickness error of the dielectric layer obtained through PECVD is about a few nanometers according to [36,37], which has little effect on the in-plane coupling efficiency. Therefore, the additional Si_3_N_4_ layer over the grating, with desired thickness at a certain position over the grating, can be easily fabricated through CMP and PECVD processes, ensuring that the idea of CE enhancement by Fabry-Perot-like cavity is feasible in the lab.

**Figure 9:**
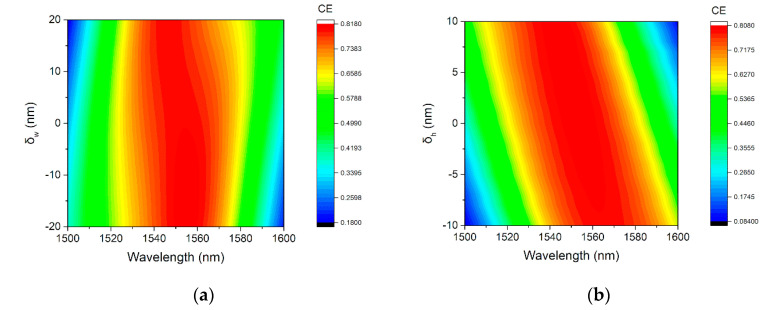
(**a**) Dependence of CE on width errors of the grating tooth. (**b**) Dependence of CE on grating etch depth errors.

To investigate the robustness of our optimized devices, four sets of studies were performed that represent commonly encountered fabrication errors: variation of the grating width, of the etch depth, of the thickness of the deposited Si_3_N_4_ layer, and of the cavity distance. In Figure 9a, we plot the CE for grating tooth width variation, *δ_w_* varies in the range of ±20 nm. As seen, over a variation of ±20 nm, the CE changes are small. In Figure 9b, we repeat the same analysis for variation of etch depth *δ_h_*. Such deviations are expected to be within ±10 nm [38]. A CE of >70% can be maintained around 1550 nm for deviations of <±10 nm. The CE is more sensitive to the variation of *δ_h_*, since etch depth plays an important role in the phase matching condition of the up-diffracted wave for Si-overlay GCs.

**Figure 10:**
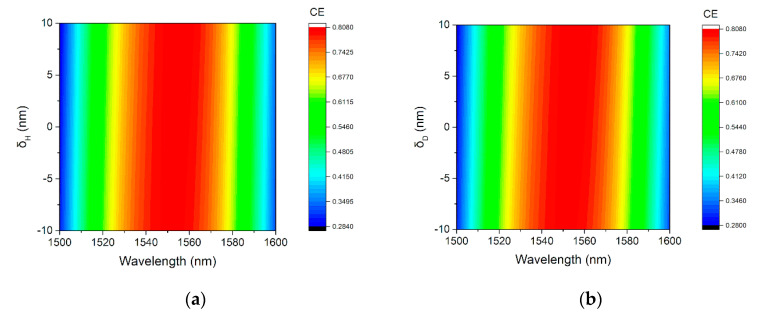
Fabrication tolerances of the CE: (**a**) Thickness error of the Si_3_N_4_ layer. (**b**) Thickness error of the SiO_2_ between grating and Si_3_N_4_ layer.

Finally, in Figure 10, we show an additional analysis, where variations of the thickness of the Si_3_N_4_ layer *δ_H_* and of the cavity distance *δ_D_* are considered. According to Figure 10, little degradation of the CE is expected. Therefore, subjected to fabrication variations, the CE profile for the proposed GC is quite robust.

### 4.2. Tolerance of the Polarization Angle

**Figure 11:**
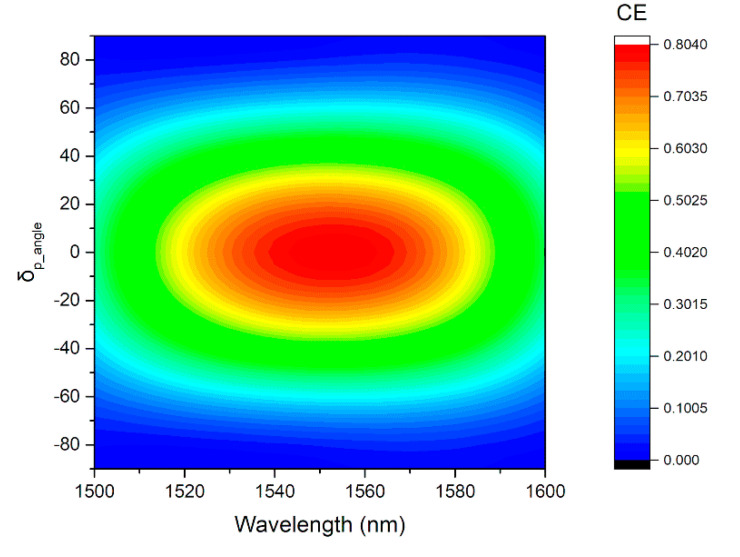
Dependence of coupling efficiency on deviation of the angle of polarization δ_p_angle._

The grating coupler proposed here is designed to couple transverse-electric (TE) polarized light into on-chip waveguides, since lots of SOI waveguide devices operate in quasi TE mode. Therefore, the coupling efficiency of our coupler is quite poor for transverse-magnetic (TM) polarized light. As shown in Figure 11, the CE can be maintained above 70% when the deviation of the polarization angle δ_p_angle_ varies from −20 degree to +20 degree (the polarization angle for TE polarized light is 0 degree), but drops to nearly 1% when the polarization angle reaches −90 degree or +90 degree, which corresponds to TM polarized light. So far, we have been focusing on improving the performance of the grating coupler, considering TE polarization. The coupling of TM polarized light will be studied in future works.

## 5. Conclusions

In conclusion, we have presented a broadband high-efficiency perfectly vertical GC, with minimum feature size larger than 219 nm. By taking advantage of an additional Si_3_N_4_ layer and the idea of Si-overlay for a GC, the peak CE reaches up to 80.3%, with up-reflection below 5%. The Si_3_N_4_ layer over the GC acts as a reflector and forms a Fabry-Perot-like cavity combined with the GC. With the help of the cavity, the light back-reflected towards the launching fiber is suppressed, thus enhancing the coupling into on-chip waveguides. In addition, since there are fewer grating periods, the proposed GC shows a wide-band character with a 1-dB bandwidth of 58 nm, ranging from 1522 to 1580 nm. The minimum feature size of the designed device is over 219 nm, which makes our design more cost-effective compared to other GC demonstrations. Moreover, our design maintains these high levels of CE even when subjected to typical fabrication variations, including grating width, etch depth, and deposition variation. Our perfectly vertical GC is believed to be suitable for efficient and fabrication-tolerant interfacing application between off-chip light sources and integrated chips that can be mass-produced.

## Figures and Tables

**Table 1:** Main parameters used in simulations at 300 K.

Waveguide Thickness (μm)	0.22
Buried Oxide Thickness (μm)	2
Si-overlay Thickness (μm)	0.16
Grating Groove Depth (μm)	0.23
Grating Periods Number	17
Si_3_N_4_ Refractive Index (λ = 1.55 µm)	2.03 ^a^
Fiber Gaussian Mode Waist Radius (μm)	5.2
Fiber Gaussian Mode Polarization Angle (deg)	90

^a^ From Ref [32].
